# Differential Roles of IL-18 and IL-8 Gene Variations in Multiple Sclerosis: Associations with Susceptibility and MRI Disease Activity

**DOI:** 10.3390/jcm15093281

**Published:** 2026-04-25

**Authors:** Sezgin Kehaya, Arzu Ay, Nevra Alkanli, Ayse Nur Cesme

**Affiliations:** 1 Department of Neurology, Faculty of Medicine, Trakya University, Edirne 22030, Turkey; 2 Department of Biophysics, Faculty of Medicine, Trakya University, Edirne 22030, Turkey; arzuay@trakya.edu.tr (A.A.); anurcesme@icloud.com (A.N.C.); 3 Department of Biophysics, Faculty of Medicine, Haliç University, Istanbul 34060, Turkey; nevraalkanli@halic.edu.tr

**Keywords:** multiple sclerosis, IL-18 variation, IL-8 variation, magnetic resonance imaging, neuroinflammation

## Abstract

**Background/Objectives**: Cytokine-mediated immune dysregulation contributes to the heterogeneity of multiple sclerosis (MS). Interleukin-18 (IL-18) and interleukin-8 (IL-8) are involved in distinct inflammatory pathways; however, their genetic contributions to disease susceptibility and radiological activity remain incompletely defined. **Methods**: In this study, 98 relapsing–remitting MS (RRMS) patients and 98 healthy controls were genotyped for IL-18 and IL-8 variations using PCR-based methods. Clinical data and MRI findings were analyzed in the MS cohort. Associations with disease susceptibility, clinical severity (EDSS), and MRI activity were evaluated using regression analyses. **Results**: IL-18 (−137 G/C) and IL-8 variations were significantly associated with MS susceptibility. The G allele of IL-18 (−137), the T allele of IL-8 (−251), and the C allele of IL-8 (+781) were more frequent in MS patients. No significant associations were observed between cytokine variations and clinical severity measures. However, IL-18 (−137) variation was significantly associated with higher baseline MRI lesion burden, with C allele carriers showing increased lesion counts. In addition, IL-8 (−251 AA genotype) was independently associated with increased annual lesion development. These findings were confirmed in multivariable regression analyses. **Conclusions**: IL-18 and IL-8 gene variations contribute to MS through distinct but complementary mechanisms. IL-18 appears to be primarily involved in disease susceptibility and baseline inflammatory burden, whereas IL-8 is more closely associated with ongoing radiological activity. These results highlight the importance of integrating genetic and imaging biomarkers to better understand disease heterogeneity in MS.

## 1. Introduction

Multiple sclerosis (MS) is a chronic autoimmune disorder targeting the central nervous system, defined by the destruction of myelin and progressive neuroaxonal damage. Relapsing-remitting MS (RRMS), the most prevalent early clinical phenotype, is primarily characterized by localized inflammatory episodes; however, it encompasses a diverse biological spectrum that varies significantly across individual patients [1].

Cytokine and chemokine pathways contribute to this heterogeneity by regulating immune activation, cell migration, and tissue injury. Within this inflammatory network, interleukin-8 (IL-8) and interleukin-18 (IL-18) represent two relevant but distinct components. IL-8 is primarily associated with leukocyte recruitment, intrathecal inflammation, and early inflammatory disease activity, with increased CSF levels linked to radiological activity and unfavorable clinical course [2,3]. In contrast, IL-18, a member of the IL-1 cytokine family, acts as a potent inducer of IFN-γ and amplifies Th1-type immune responses through inflammasome-related pathways [4].

Increased levels of IL-18 have been documented in the serum and cerebrospinal fluid of MS patients, notably during clinical exacerbations, where they serve as markers for both disease activity and long-term progression. Promoter variations in the IL18 gene, especially −137 G/C and −607 C/A, may influence transcriptional activity and have been investigated as susceptibility factors, although results vary across populations [5,6]. Importantly, several studies have evaluated IL-18 variations together with serum IL-18 levels, suggesting that genetic variation and cytokine expression should be interpreted in combination rather than independently [7,8].

For IL-8, available studies have largely focused on cytokine levels and their association with inflammatory activity, radiological reactivation, and disease evolution, supporting a role in active lesion biology rather than long-term progression alone [2,3]. However, compared with IL-18, the genetic aspects of IL-8 in MS have been less extensively investigated.

Despite these findings, an important gap remains. Most previous studies have examined either cytokine levels or genetic susceptibility separately, while data integrating cytokine gene variations with objective MRI-defined disease burden are limited. This is particularly relevant in RRMS, where radiological activity may better reflect inflammatory disease expression than clinical disability alone.

Therefore, in this study, we aimed to investigate the association of IL-8 and IL-18 gene variations with MS in two steps: first, by evaluating their relationship with disease susceptibility through comparison of RRMS patients and healthy controls, and second, by assessing their association with clinical and radiological disease features within the RRMS cohort, with particular emphasis on MRI lesion burden and lesion dynamics during follow-up.

## 2. Materials and Methods

This investigation was conducted in compliance with the Declaration of Helsinki following approval by the Trakya University Faculty of Medicine Non-Interventional Scientific Research Ethics Committee (Approval No: TÜTF-GOBAEK 2023/150, Decision: 06/16, Date: 10 April 2023. Each participant gave their written informed consent prior to their inclusion in the study cohort. Based on the study conducted by Orhan G et al., titled “The association of IL-18 gene promoter polymorphisms and the levels of serum IL-18 on the risk of Multiple Sclerosis,” the required sample size for the present study was determined using a power analysis. With a Type I error rate (alpha) of 0.05 and a statistical power (1-beta) of 0.95, it was calculated that a minimum of 88 participants per group (total N = 176) is necessary to detect significant differences between the groups. Patients diagnosed with RRMS according to the 2017 revised McDonald criteria and healthy volunteers were invited to participate. Patients younger than 18 years of age, those without available MRI data, and those with concomitant inflammatory or systemic diseases were excluded. Only patients with at least 1 year of clinical follow-up and a minimum of two MRI examinations were included. Participants who met the inclusion criteria were enrolled consecutively, and peripheral venous blood samples were obtained at baseline. Demographic and baseline clinical data were collected in accordance with established MS cohort methodologies, including age, sex, smoking status, and comorbid conditions such as hypertension, diabetes mellitus, dyslipidemia, and coronary artery disease, as well as disease duration and clinical characteristics at disease onset [2,3]. At the time of enrollment, the patient cohort exhibited clinical heterogeneity regarding treatment status, including treatment-naive individuals and those receiving various first-line or second-line disease-modifying therapies (DMTs). Decisions regarding the initiation or switching of DMTs were made by treating neurologists based on standard clinical guidelines and disease activity.

Peripheral venous blood leukocytes were utilized for genomic DNA extraction via column-based isolation techniques, following established protocols. The quality and yield of the extracted DNA were rigorously evaluated by assessing purity and concentration levels before proceeding with the molecular analysis [6,9]. The study focused on IL-18 promoter variations (−137 G/C [rs187238] and −607 C/A [rs1946518]) and IL-8 variations (−251 A/T and +781 C/T), selected based on their potential regulatory effects on cytokine expression and inflammatory responses. Genotyping was performed using polymerase chain reaction (PCR) and restriction fragment length polymorphism (RFLP) methods. PCR mixtures consisted of template DNA, reaction buffer, dNTPs, sequence-specific primers, Taq DNA polymerase, and MgCl_2_. For IL-18 −137 G/C variation, forward allele-specific primers (5′-CCCCAACTTTTACGGAAGAAAAG-3′ and 5′-CCCCAACTTTTACGGAAGAAAAC-3′), a control primer, and a reverse primer were used, with thermal cycling conditions consisting of an initial denaturation at 94 °C for 3 min, followed by 40 cycles of denaturation at 94 °C for 20 s, annealing at 54 °C for 20 s, and extension at 72 °C for 20 s, with a final extension at 72 °C for 5 min. For IL-18 −607 C/A variation, allele-specific primers were used under similar conditions with an annealing temperature of 50 °C. For IL-8 −251 A/T variation, amplification was performed using forward and reverse primers (5′-CCATCATGATAGCATCTGT-3′ and 5′-CCACAATTTGGTGAATT-3′) under thermal cycling conditions including an initial denaturation at 94 °C for 5 min, followed by 35 cycles of denaturation at 94 °C for 30 s, annealing at 56 °C for 30 s, and extension at 72 °C for 1 min, with a final extension at 72 °C for 8 min. PCR products were subsequently digested using the VspI (AseI) restriction enzyme to determine genotype distribution. For IL-8 +781 C/T variation, PCR amplification was performed using sequence-specific primer pairs followed by EcoRI restriction enzyme digestion for genotype determination. Amplification products were visualized using agarose gel electrophoresis, and genotype assignments were made based on fragment size patterns. All genetic analyses were performed after completion of clinical and radiological data collection, and laboratory personnel were blinded to patient characteristics. Genotyping was performed for all participants, including 98 MS patients and 98 controls (total n = 196). To ensure the highest technical rigor and data integrity, we implemented strict quality control (QC) metrics. The overall genotype call rate was 100% for all four SNPs, with no missing data across the entire cohort. To validate the accuracy of the genotype calls, 10% of the samples were randomly selected and re-genotyped, achieving a 100% concordance rate. Hardy–Weinberg equilibrium (HWE) was assessed for each variation in both groups using the Chi-square (χ^2^) test. Primary genetic association analyses were conducted using a dominant model, where variant allele carriers were grouped together: GC+CC vs. GG for IL-18 −137, CA+AA vs. CC for IL-18 −607, AT+TT vs. AA for IL-8 −251, and CT+TT vs. CC for IL-8 +781. The dominant model was chosen to maintain statistical power and focus on the impact of the presence of the risk allele.

Clinical severity was assessed using the Expanded Disability Status Scale (EDSS) at baseline and during follow-up, in accordance with established clinical evaluation approaches [3]. Disease duration was defined as the time from first symptom to study inclusion. Additional clinical variables included age at first symptom, time interval from first symptom to diagnosis, time required to reach an EDSS score of 3, which was used as an indicator of disability progression, and annualized relapse rate during the follow-up period. Clinical presentation at disease onset was classified according to the initial neurological involvement as optic, brainstem, spinal, or supratentorial. Previous clinical records were reviewed to ensure accurate characterization of disease onset and progression, and follow-up evaluations over the 12-month period were used to assess the clinical course.

All patients underwent brain magnetic resonance imaging (MRI) using a 1.5 Tesla scanner with a standardized protocol. MRI acquisition and evaluation were performed in accordance with established MS imaging approaches [2,3]. Baseline MRI evaluation included assessment of total T2-weighted lesion count, lesion localization, and presence of gadolinium-enhancing lesions. Lesion localization was categorized according to anatomical regions, including spinal involvement (cervical or thoracolumbar) and cerebral involvement, which was further classified as periventricular, juxtacortical, infratentorial, or optic. Follow-up MRI examinations were evaluated for radiological disease activity, defined as the presence of new T2 lesions, enlargement of existing lesions, or gadolinium-enhancing lesions. Lesion number and localization were determined based on radiology reports and confirmed by the neurologist’s review.

### Statistical Analysis

Data analysis was conducted using IBM SPSS Statistics version 25 (Armonk, NY, USA). We assessed the distribution of continuous variables via the Kolmogorov–Smirnov test; normally distributed data are reported as mean ± SD, while non-parametric data are presented as median (IQR). Categorical data are expressed as absolute numbers and percentages. Genotype and allele frequencies were compared using the chi-square test or Fisher’s exact test, and Hardy–Weinberg equilibrium was assessed for all variations. The relationship between genetic variants and continuous outcomes was evaluated using either Student’s *t*-test or the Mann–Whitney U test, as appropriate for the data distribution. Logistic regression analysis was used to identify independent predictors of high baseline lesion burden, defined as values above the cohort median, adjusting for age, sex, and disease duration. Linear regression analysis was performed to evaluate factors associated with lesion development during follow-up. Multicollinearity was assessed prior to regression analyses. A *p*-value < 0.05 was considered statistically significant. Due to the exploratory nature of this study and our focus on pre-selected candidate cytokine genes, nominal *p*-values were reported without adjustment for multiple comparisons. This approach was adopted to maximize the identification of potentially relevant genetic markers for MS, acknowledging the increased risk of Type I error.

## 3. Results

### 3.1. Demographic Characteristics of the Study Population

Demographic and variation characteristics are summarized in Table 1. Most baseline variables were comparable between MS patients and controls. Smoking was more frequent in the MS group (*p* = 0.040). The prevalence of hypertension, diabetes mellitus, dyslipidemia, and coronary artery disease did not differ significantly between groups. Regarding cytokine variations, significant differences in genotype distributions were observed for IL-18 (−137 G/C), IL-8 (−251 A/T), and IL-8 (+781 C/T), while IL-18 (−607 C/A) showed no significant difference between MS patients and controls.

### 3.2. Association Between Cytokine Variations and MS Susceptibility

The associations between cytokine gene variations and MS susceptibility are summarized in Table 2, while genotype distributions for all loci are provided in Table 1. Allele frequency analysis revealed significant differences between patients and controls for several loci. Specifically, the IL-18 (−137 G/C) and IL-8 variations (−251 A/T and +781 C/T) were significantly associated with MS susceptibility, whereas IL-18 (−607 C/A) showed no significant difference between groups. Allele frequencies were derived from genotype distributions (e.g., GG, GC, CC or TT, CT, CC) and therefore represent the overall contribution of each allele within the study population. In this analysis, the G allele of IL-18 (−137), the T allele of IL-8 (−251), and the C allele of IL-8 (+781) were more frequent in MS patients, suggesting a potential contribution of these variants to disease susceptibility. The distribution of IL-18 (−137 G/C) genotypes showed a highly significant difference between MS patients and controls (*p* = 0.0009), derived from a comprehensive genotype-based comparison (AA vs. CA vs. CC). Due to the small number of homozygous variant carriers (CC, n = 4), Fisher’s Exact Test was employed to ensure statistical rigor. Although formal multiple testing corrections were not primarily applied due to the exploratory nature of the study, this specific association is remarkably robust, as the *p*-value remains well below the conservative Bonferroni-adjusted threshold (*p* < 0.0125). This indicates that the IL-18 (−137) variation serves as a strong genetic marker for MS susceptibility in our cohort, rather than being a chance observation stemming from small subgroup sizes.

Allele frequencies were derived from genotype distributions. Odds ratios (OR) and 95% confidence intervals (CI) were calculated to assess the association between cytokine gene variations and MS susceptibility. The less frequent allele in controls was used as the reference.

### 3.3. Hardy–Weinberg Equilibrium

Technical reliability was confirmed by a 100% call rate for all variations in both patients and controls. To evaluate the reliability of genotype distributions and detect potential population stratification or genotyping bias, HWE analysis was performed for each variation in both MS patients and controls. HWE assesses whether the observed genotype frequencies correspond to the expected distribution in a genetically stable population. For IL-18 (−137 G/C), genotype distributions deviated from HWE in the MS group (χ^2^ = 4.77, *p* = 0.029) but were consistent with equilibrium in the control group (χ^2^ = 2.13, *p* = 0.144). For IL-18 (−607 C/A), deviations from HWE were observed in both MS patients (χ^2^ = 4.44, *p* = 0.035) and controls (χ^2^ = 4.41, *p* = 0.036). The IL-8 (−251 A/T) variation was consistent with HWE in the MS group (χ^2^ = 1.37, *p* = 0.241), whereas a deviation was detected in controls (χ^2^ = 5.04, *p* = 0.025). Finally, IL-8 (+781 C/T) showed significant deviation from HWE in both MS patients (χ^2^ = 49.93, *p* < 0.001) and controls (χ^2^ = 27.56, *p* < 0.001). Despite these statistical deviations, the 100% concordance rate from re-genotyping validation and the absence of any failed genotype calls suggest that these findings are not attributable to technical errors. Instead, the observed patterns—particularly the strong deviations in IL-8 (+781 C/T)—likely reflect biological factors such as population stratification, selection bias, or the inherent genetic structure of the study population.

### 3.4. Clinical and Radiological Associations of Cytokine Variations in the MS Cohort

Within the MS cohort, the median EDSS score was 3.0 (IQR 2.0–4.0) with a median disease duration of 7 years (IQR 3–12). The median time from symptom onset to diagnosis was 24 months (IQR 12–48). Patients experienced a median annual relapse rate of 0.8 attacks per year (IQR 0.4–1.2), and the median MRI lesion count at diagnosis was 11 (IQR 6–18). Associations between cytokine gene variations and clinical disease severity markers, including EDSS score, time to EDSS ≥ 3, and annual relapse rate, were subsequently evaluated. Overall, no statistically significant relationships were identified between the investigated variations and clinical severity measures. However, analysis of the IL-18 (−137 G/C) variation revealed a trend toward differences in disability progression, with carriers of the GC genotype demonstrating a longer time to reach EDSS ≥ 3 compared with other genotype groups (*p* = 0.07). Similarly, patients with the IL-8 (−251 AA genotype) tended to have slightly higher EDSS scores, although this difference did not reach statistical significance. No meaningful associations were observed for IL-18 (−607 C/A) or IL-8 (+781 C/T) variations with the evaluated clinical severity parameters. The associations between cytokine gene variations and the clinical presentation of MS were also examined using initial symptom type, symptom at diagnosis, and time to diagnosis, as summarized in Supplementary Table S1A,B. No statistically significant relationships were detected between the investigated variations and the distribution of clinical symptoms at disease onset or diagnosis. Optic and supratentorial presentations represented the most frequent clinical manifestations across genotype groups, and diagnostic delay did not significantly differ between the evaluated variation groups. Clinical analyses were further extended to evaluate whether cytokine gene variations were associated with the localization of the first new lesion after diagnosis. The most frequent locations of the first post-diagnostic lesion were cervical spinal cord, periventricular, and juxtacortical regions. However, no statistically significant associations were observed between cytokine variations and lesion localization for IL-18 (−607 C/A), IL-18 (−137 G/C), IL-8 (−251 A/T), or IL-8 (+781 C/T) variants. In addition, we evaluated whether cytokine gene variations were associated with the localization of the first lesion after diagnosis. Lesion locations were grouped as spinal (cervical or thoracolumbar) and cerebral (periventricular, juxtacortical, infratentorial, or optic). Among MS patients, 55 lesions were cerebral and 43 were spinal. However, no statistically significant associations were observed between cytokine variations and lesion localization for IL-18 (−607 C/A), IL-18 (−137 G/C), IL-8 (−251 A/T), or IL-8 (+781 C/T) variants. Nevertheless, a numerical tendency toward a higher proportion of spinal lesions was observed among carriers of the IL-8 (−251 AA genotype) (6 spinal vs. 5 cerebral lesions), although this difference did not reach statistical significance.

### 3.5. Cytokine Variations and MRI Findings

MRI analyses demonstrated significant associations between cytokine variations and radiological disease activity (Table 3). A significant difference in baseline lesion burden was observed for the IL-18 (−137 G/C) variation. Patients carrying the CC genotype exhibited a higher lesion count at diagnosis compared with GC and GG genotypes (median 17 vs. 11 lesions, *p* = 0.041). In addition, the IL-8 (−251 A/T) variation was significantly associated with annual lesion development (*p* = 0.047), with AA genotype carriers demonstrating higher rates of new lesion formation during follow-up. No significant associations were observed between IL-18 (−607 C/A) or IL-8 (+781 C/T) variations and MRI lesion burden. To further explore these findings, dominant genetic models were applied. Carriers of the IL-18 (−137) C allele (GC+CC) showed a significantly higher lesion burden at diagnosis compared with GG genotype carriers (*p* = 0.038), whereas no significant differences were observed for the other investigated variations. Overall, these findings suggest that the IL-18 (−137) variation may influence baseline inflammatory lesion burden, whereas the IL-8 (−251) variation may be associated with ongoing radiological disease activity during follow-up.

To determine whether the observed genetic associations were independent predictors of MRI disease activity, multivariable regression analyses were performed (Table 4). Logistic regression analysis was used to evaluate predictors of high baseline lesion burden, defined as a lesion count greater than or equal to the cohort median. After adjustment for age, sex, and disease duration, carriage of the IL-18 (−137) C allele (GC+CC) remained independently associated with increased baseline MRI lesion burden. In addition, linear regression analysis was performed to assess factors associated with annual lesion development. In this model, the IL-8 (−251 AA genotype) emerged as an independent predictor of increased annual lesion formation during follow-up. No other investigated cytokine variations demonstrated independent associations with MRI disease activity.

**Figure 1:**
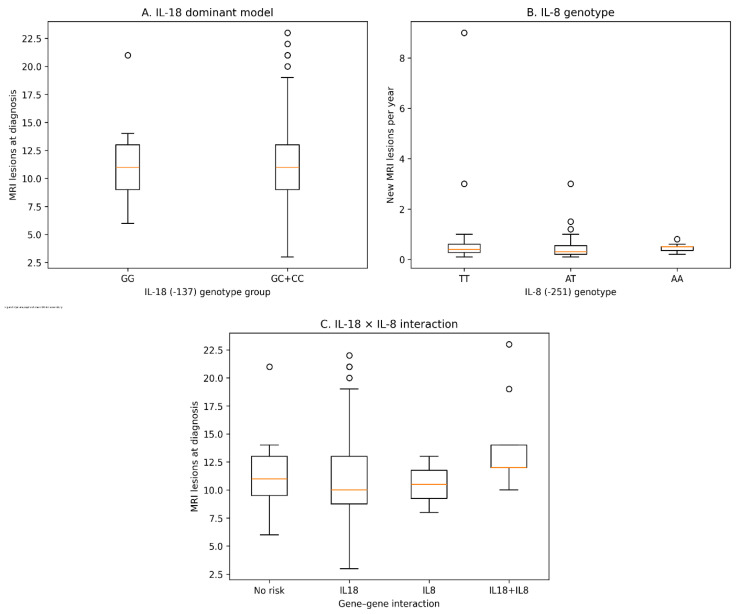
Associations between cytokine gene variations and MRI disease activity in RRMS. (**A**) Baseline MRI lesion burden according to the IL-18 (−137 G/C) dominant model. (**B**) Annual new MRI lesion development according to IL-8 (−251 A/T) genotype. (**C**) Gene–gene interaction analysis demonstrating combined effects of IL-18 (−137) and IL-8 (−251) variants on MRI lesion burden.

To visually illustrate the observed genetic effects on MRI disease activity, boxplot analyses were performed for both baseline lesion burden and annual lesion development (Figure 1). Consistent with the regression analyses, carriers of the IL-18 (−137) C allele demonstrated higher baseline lesion counts compared with GG genotype carriers. In addition, patients with the IL-8 (−251 AA genotype) showed increased rates of new lesion formation during follow-up. A combined gene–gene interaction analysis further suggested a tendency toward higher lesion burden among individuals carrying both IL-18 and IL-8 risk variants, although this interaction did not reach statistical significance.

## 4. Discussion

In this study, we investigated the role of IL-8 and IL-18 gene variations in RRMS, focusing on disease susceptibility, clinical characteristics, and radiological activity. Our findings indicate that IL-18 variations are more strongly associated with MS susceptibility, whereas IL-8 appears to be more closely related to inflammatory activity and disease expression. These results support the concept that cytokine-mediated immune dysregulation plays a central role in MS pathogenesis, involving complex interactions between innate and adaptive immune pathways [10].

The role of IL-18 in MS susceptibility has been demonstrated across multiple populations. In our cohort, IL-18 variations were associated with disease risk, which is consistent with previous findings in Turkish populations, where the −137 CC genotype was significantly associated with MS and conferred an increased risk [6,11]. Similarly, studies in Middle Eastern and European populations have reported associations between IL-18 promoter variations, cytokine expression levels, and MS susceptibility, supporting a population dependent but biologically consistent role for IL-18 [7,8]. Another study has shown that IL-18 promoter variants, particularly −607C/A, significantly increase susceptibility, highlighting the importance of regulatory variations in disease development [6]. In addition, gene expression studies demonstrated that IL-18 expression is significantly elevated in MS patients compared to controls, further supporting its biological relevance [12]. Mechanistically, IL-18 is a key cytokine bridging innate and adaptive immunity by promoting IFN-γ production and Th1 polarization. It is produced by macrophages, dendritic cells, and microglia, and contributes to the amplification of inflammatory cascades within the central nervous system [4,13]. Importantly, IL-18 promoter variations have been shown to influence transcriptional activity through modification of transcription factor binding sites, resulting in altered cytokine expression and inter-individual differences in cytokine production [5,14]. This supports the hypothesis that genetic variation affects immune regulation rather than simply increasing inflammation. Indeed, cytokine imbalance, rather than absolute cytokine levels, appears to drive immune dysregulation in MS [15]. Furthermore, IL-18 contributes to T-cell activation and immune regulation, reinforcing its role in MS pathogenesis [16]. Recent studies have also highlighted the role of IL-18 within inflammasome pathways. The NLRP3 inflammasome has emerged as a key driver of neuroinflammation in MS, linking innate immune activation to cytokine release, including IL-18 [1,17]. In addition, mitochondrial dysfunction has been shown to amplify inflammasome activation and inflammatory signaling, further contributing to neurodegeneration [18]. These findings indicate that IL-18 plays a central role not only in disease susceptibility but also in ongoing inflammatory and neurodegenerative processes. Consistent with these mechanisms, elevated IL-18 levels have been detected in serum and cerebrospinal fluid (CSF) of MS patients, particularly during active disease phases and in association with radiological activity [14,19,20]. Moreover, IL-18 has been associated with immune cell activation, including CD8+ T cell subsets involved in CNS infiltration, highlighting its role in both peripheral and central immune responses [21].

In contrast to IL-18, IL-8 appears to play a more prominent role in disease activity rather than susceptibility. IL-8 is a chemokine involved in leukocyte recruitment and blood–brain barrier disruption, and its levels are closely associated with inflammatory activity in MS. Elevated IL-8 levels in CSF have been linked to intrathecal inflammation, disease activity, and worse clinical outcomes [2,3]. Furthermore, IL-8 has been identified as a predictor of conversion from clinically isolated syndrome to MS, highlighting its role in early disease dynamics [22]. Differences between CSF and serum IL-8 levels suggest compartmentalized immune activation within the central nervous system [2,23]. Elevated CSF IL-8 levels were associated with disease progression, too [24]. Additionally, IL-8 has been linked to virus-driven immune responses, particularly Epstein–Barr virus-related immune activation, which is a well-established environmental risk factor for MS. Increased IL-8 responses in the CNS have been associated with EBV-related immune activation, suggesting that IL-8 may act as a mediator of environmentally triggered inflammation [25].

Interestingly, our study did not demonstrate a strong association between cytokine variations and clinical disability measures such as EDSS. This observation may be explained by the concept of clinicoradiological dissociation in MS. While inflammatory activity is reflected by MRI lesion burden, clinical progression is largely driven by neurodegenerative processes, including axonal loss and mitochondrial dysfunction. In this context, MS has been proposed as a length-dependent central axonopathy in which inflammatory and neurodegenerative processes evolve asynchronously [26,27]. According to the “therapeutic lag” hypothesis, suppression of inflammation does not immediately translate into clinical stabilization, which may explain why cytokine-related genetic variation is associated with radiological activity but not clinical disability. Therefore, cytokine-related genetic variations may primarily influence inflammatory activity rather than long-term disability.

In contrast, we observed clearer associations with radiological disease activity. MRI-based studies have shown that inflammatory processes are closely linked to lesion formation, chronic active lesions, and ongoing neuroinflammation [28]. IL-18 may contribute to early lesion formation through Th1-mediated mechanisms, whereas IL-8 may be more relevant in sustaining inflammatory activity within established lesions. This temporal distinction provides a novel perspective on cytokine-driven disease mechanisms.

Inconsistencies observed between our results and those of earlier research could be attributed to distinct population-specific genetic architectures, diverse environmental factors, and discrepancies in methodological frameworks. Increasing evidence suggests that interactions between genetic susceptibility and environmental factors, including infections and microbiota composition, play a crucial role in MS pathogenesis [29,30].

The primary significance of this research stems from the synergistic analysis of genetic, clinical, and neuroimaging parameters within a prospective framework. Such a multi-dimensional strategy facilitates a more nuanced understanding of genotype-phenotype correlations and elucidates the specialized functions of cytokines throughout the disease course. By delineating the unique impacts of IL-18 and IL-8 on MS susceptibility and clinical activity, our findings offer a novel perspective that reinforces the theory of temporal heterogeneity in the pathology of MS.

It is important to note that the identified genetic variations in IL-8 and IL-18 do not directly prove a functional change in protein expression or activity. While these variations are statistically linked to MS susceptibility in our cohort, our study did not measure systemic or local cytokine levels. Therefore, any mechanistic link between the observed genotypes and MS pathogenesis remains speculative. Genetic variations often exert their effects through complex regulatory networks, and the lack of protein data limits our ability to draw definitive conclusions about the functional impact of these SNPs. Our results should be viewed as a genetic framework that requires further validation through functional assays and protein expression profiling.

Nevertheless, this study is subject to certain limitations. Primarily, the relatively modest sample size might constrain the statistical power of our findings, especially when analyzing genotypes with lower allelic frequencies. In alignment with this exploratory framework, we reported our findings based on nominal *p*-values rather than applying stringent multiple testing corrections, such as the False Discovery Rate (FDR). Given the complex polygenic architecture of Multiple Sclerosis and our targeted focus on candidate genes with established roles in neuroinflammation, this methodological choice was deliberately made to avoid the loss of potentially meaningful biological signals (Type II error).

While prioritizing sensitivity ensures that suggestive associations in these inflammatory pathways remain available for future large-scale replication and meta-analyses, we acknowledge that this approach inherently increases the risk of Type I errors (false positives). Consequently, the absence of multiple testing corrections remains a significant statistical limitation of our study. These results should therefore be interpreted as preliminary, hypothesis-generating data rather than definitive conclusions. Future research involving larger, independent cohorts is strictly required to validate these associations and to resolve the potential for inflated significance levels in the context of MS pathogenesis.

Beyond these statistical constraints, certain biological and clinical factors should also be considered when interpreting our results. Primarily, the absence of cytokine level measurements prevents direct correlation between genetic variation and protein expression, leaving the functional consequences of the identified polymorphisms to be elucidated in future functional assays. Additionally, the follow-up duration of this cohort may not fully capture the nuances of long-term disease progression and clinical disability accumulation. Another limitation of our study is the significant deviation from Hardy–Weinberg Equilibrium (HWE) observed in certain loci, particularly IL-8 (+781 C/T). While strong HWE deviations can sometimes be associated with genotyping errors, our study maintained a 100% genotype call rate and achieved 100% concordance in re-genotyping validation, suggesting that technical artifacts are not the cause. Instead, these deviations may be attributed to our relatively small sample size or population stratification within the study cohort. Although we recruited participants from the same geographical region to ensure homogeneity, cryptic ancestral sub-structures can lead to such imbalances. Future studies with larger, multi-center cohorts are needed to confirm these genetic distributions and further explore the potential selection bias in these cytokine gene clusters. Another limitation of our study is the small sample size in certain genotype subgroups, such as the IL-18 (−137) CC genotype (n = 4). Statistical analyses involving such small subgroups can be unstable and may lead to inflated effect sizes or restricted generalizability. However, it is noteworthy that the association observed for this locus was highly significant (*p* = 0.0009), suggesting a robust genetic signal that persists even under stringent post hoc thresholds, such as a Bonferroni-adjusted alpha (*p* < 0.0125). Consequently, while the findings related to these specific low-frequency genotypes should be interpreted with caution and regarded as preliminary, the statistical strength of the association indicates that they are unlikely to be mere stochastic artifacts. Nevertheless, larger cohorts are required to confirm the clinical significance and reproducibility of these rare genotype distributions in MS patients. Finally, our study is characterized by its exploratory design. We did not apply formal statistical corrections (e.g., FDR or Bonferroni) for multiple testing. While this increases the probability of identifying nominal associations, it ensures that suggestive genetic signals are not overlooked in this pilot phase. We recommend that our findings be interpreted as preliminary and should be validated in independent, larger-scale studies using more stringent adjustment thresholds. The absence of functional data, such as serum cytokine levels or mRNA expression profiles, prevents us from making definitive mechanistic claims. The biological significance of the observed genetic associations remains to be fully elucidated through future functional studies. A further limitation of this study is the lack of adjustment for potential confounding variables such as smoking status, treatment history, and comorbidities in the regression models. Although we adjusted for age, sex, and disease duration, smoking status was found to differ significantly between groups (*p* = 0.040). Since smoking and specific disease-modifying therapies (DMTs) are known to modulate cytokine profiles and clinical outcomes in MS, their exclusion may affect the observed genetic associations. Consequently, our findings should be considered preliminary, and the independent effect of IL-8 and IL-18 variations remains to be confirmed in models that fully account for these environmental and clinical parameters. Furthermore, our inclusion criteria—requiring at least one year of clinical follow-up and a minimum of two MRI scans—may have introduced a potential selection bias. While these criteria were necessary to ensure the quality and consistency of the longitudinal data, they might have resulted in the exclusion of patients at the extreme ends of the disease spectrum, including those with very mild symptoms who required less frequent monitoring or those with highly aggressive disease courses. Consequently, the genetic associations observed in this study should be interpreted as being representative of a specific subgroup of MS patients, and their generalizability to the broader MS population requires validation in cohorts with more diverse follow-up profiles. Another limitation is the potential inclusion of patients with co-existing minor or stable autoimmune conditions, such as autoimmune thyroiditis or past episodes of other immune-mediated problems. While our exclusion criteria focused on active, systemic inflammatory diseases to maintain cohort homogeneity, the presence of subclinical or controlled autoimmune issues cannot be entirely ruled out. Given that genetic variations in cytokine genes like IL-18 and IL-8 are often shared across various autoimmune pathologies, the presence of these comorbidities might have influenced the observed associations. Future research should include a more granular assessment of the patients’ full autoimmune history to better isolate MS-specific genetic effects. Furthermore, the assessment of new radiological activity did not account for variations in treatment regimens, including the administration of steroid boluses during relapses or the use of different disease-modifying therapies (DMTs). Since these treatments can significantly suppress the formation of new inflammatory lesions, the radiological outcomes reported here should be interpreted as reflecting the disease course under standard clinical management rather than a natural, untreated progression. The heterogeneity of treatments within our cohort precluded a robust stratified analysis; however, this remains an essential factor for future prospective studies to control for when evaluating the predictive value of IL-8 and IL-18 genotypes on MRI outcomes. The diversity in treatment exposure (first-line vs. second-line DMTs) represents an analytical challenge. Different classes of DMTs have distinct mechanisms of action that can modulate systemic cytokine levels and suppress MRI activity to varying degrees. Our study did not primarily adjust for the specific line of therapy, which may potentially mask or amplify certain genetic associations. While our findings reflect genetic predispositions in a real-world clinical setting, future studies with more homogeneous treatment groups or larger cohorts allowing for treatment-stratified analysis are necessary to isolate the independent effect of IL-8 and IL-18 variations from treatment-induced immunological changes. Additionally, our study lacked comprehensive data on cerebrospinal fluid (CSF) parameters, specifically the presence of oligoclonal bands (OCB). OCB status is a key marker of intrathecal inflammation and has prognostic value in MS. The absence of this information limited our ability to explore the potential correlation between IL-8/IL-18 genotypes and the intensity of the central immune response. Future research incorporating both genetic markers and CSF profiles would provide a more holistic understanding of the role of these cytokines in MS pathology.

The associations identified in this study provide a genetic framework but do not constitute direct evidence of causality or therapeutic efficacy. While the statistical links between IL-8/IL-18 variations and MS are compelling, the proposed roles of these cytokines in neuroinflammation should be viewed as hypothesis-generating observations. Future longitudinal and interventional studies are necessary to determine whether these genetic markers can indeed be translated into targeted therapeutic strategies or used to monitor disease progression.

Beyond predicting the frequency of annual relapses, it remains an open and clinically relevant question whether these genotypes can predict immediate responses to acute treatments, such as corticosteroids. Cytokines like IL-18 and IL-8 are integral to the acute inflammatory phase of MS relapses; therefore, genetic variations in these pathways might influence the speed or completeness of recovery following steroid pulse therapy. While our study did not possess the standardized clinical recovery data required for such an analysis, future prospective studies should investigate the role of IL-8 and IL-18 variations as predictive biomarkers for steroid responsiveness, which could aid in personalizing relapse management.

The association between IL-8/IL-18 variations and MS clinical parameters may have broader implications for the disease’s long-term trajectory. Emerging evidence suggests that these cytokines are not merely mediators of acute relapses but may also be involved in the critical transition from an inflammatory phenotype to a predominantly neurodegenerative one. Recent studies have highlighted how persistent low-grade inflammation driven by such innate immune signals can accelerate axonal loss and brain atrophy, which are hallmarks of progressive MS [31,32]. Furthermore, it has been demonstrated in other pathological contexts that early dysregulation of cytokine networks can predict the onset of secondary progression [33]. In this context, identifying individuals with high-risk IL-8 or IL-18 genotypes at the early stages of RRMS could provide a unique opportunity for early intervention. Targeting these pathways might not only reduce clinical relapses but could also hold the potential to delay or prevent the shift toward neurodegenerative stages, thereby preserving neurological reserve and improving long-term outcomes.

## 5. Conclusions

In conclusion, our findings suggest that IL-18 and IL-8 may act as distinct yet synergistic components within the complex neuroinflammatory cascade of Multiple Sclerosis (MS). Our data point toward IL-18 as a potential candidate factor in the genetic susceptibility and early immune-priming phase of the disease. Mechanistically, IL-18 is a crucial downstream product of inflammasome activation and a driver of Th1-mediated autoimmunity; thus, our findings suggest that its genetic variations may influence the threshold for initiating systemic pro-inflammatory responses in MS. However, the exact weight of IL-18 as a determinant in these early stages remains a hypothesis that requires further functional validation.

Conversely, the associations observed for IL-8 align with its role as a potent chemoattractant and a mediator of blood–brain barrier (BBB) integrity. The data suggest that IL-8 pathways warrant further investigation as putative markers of acute leukocyte recruitment and CNS-specific inflammatory activity. From a mechanistic perspective, IL-8 may facilitate the transmigration of immune cells across the vascular endothelium, potentially serving as a suggestive indicator of relapse dynamics and active lesion formation.

Rather than providing definitive clinical mandates, this study offers a preliminary genetic framework highlighting these cytokines as potential targets for future functional research. The apparent dichotomy between IL-18-driven priming and IL-8-mediated infiltration represents a hypothesis-generating perspective for understanding disease heterogeneity. Until validated by larger longitudinal cohorts and protein expression assays, these results should be interpreted as suggestive insights into the polygenic architecture and evolving immunopathology of MS.

## Figures and Tables

**Table 1:** Demographic characteristics, comorbidities, and genotype distributions in MS patients and controls.

Demographic and Clinical Variables	MS (n = 98)	Controls (n = 98)	*p*	Cytokine Gene Variations	MS (n = 98)	Controls (n = 98)	*p*
Age (years)	46.11 ± 12.20	45.80 ± 6.08	0.819	**IL-18 (−137 G/C)**			**0.0009**
Sex n (%) Female Male	70 (71.4) 28 (28.6)	40 (40.8) 58 (59.2)	**<0.001**	GG	77 (78.6)	54 (55.1)	
				GC	17 (17.3)	41 (41.8)	
Smoking n (%)	35 (35.7)	21 (21.4)	**0.040**	CC	4 (4.1)	3 (3.1)	
Hypertension n (%)	18 (18.4)	17 (17.3)	1.000	**IL-18 (−607 C/A)**			0.356
Diabetes mellitus n (%)	10 (10.2)	7 (7.1)	0.612	CC	73 (74.5)	64 (65.3)	
Dyslipidemia n (%)	13 (13.3)	12 (12.2)	1.000	CA	20 (20.4)	26 (26.5)	
Coronary artery disease n (%)	13 (13.3)	8 (8.2)	0.356	AA	5 (5.1)	8 (8.2)	
				**IL-8 (−251 A/T)**			**0.046**
				TT	51 (52.0)	38 (38.8)	
				AT	36 (36.7)	37 (37.8)	
				AA	11 (11.2)	23 (23.5)	
				**IL-8 (+781 C/T)**			**0.011**
				CC	44 (44.9)	24 (24.5)	
				CT	14 (14.3)	21 (21.4)	
				TT	40 (40.8)	53 (54.1)	

Values are presented as mean ± SD or n (%). Continuous variables were compared using Student’s *t*-test, and categorical variables using the chi-square test.

**Table 2:** Allele frequencies and association with MS susceptibility.

Variations	Allele	MS n (%)	Controls n (%)	OR	95% CI	*p*
IL-18 (−137)	G	171 (87.2)	149 (76.0)	2.15	1.30–3.56	**0.003**
	C	25 (12.8)	47 (24.0)	ref		
IL-18 (−607)	C	166 (84.7)	154 (78.6)	1.50	0.87–2.59	0.144
	A	30 (15.3)	42 (21.4)	ref		
IL-8 (−251)	T	138 (70.4)	113 (57.7)	1.75	1.16–2.65	**0.007**
	A	58 (29.6)	83 (42.3)	ref		
IL-8 (+781)	C	102 (52.0)	69 (35.2)	2.00	1.34–2.99	**0.001**
	T	94 (48.0)	127 (64.8)	ref		

**Table 3:** Association Between Cytokine Gene Variations and MRI Lesion Burden.

Variations	Genotype/Model	n	MRI Lesions at Diagnosis Median (IQR)	*p* Value	New MRI Lesions per Year Median (IQR)	*p* Value
**IL-18** **(−137 G/C)**	GG	77	11 (9–13)		1.2 (0.8–1.7)	
	GC	17	12 (10–16)		1.3 (0.9–1.8)	
	CC	4	17 (11–25)	**0.041**	1.4 (1.0–2.0)	0.28
	**GG vs. GC+CC** **(dominant model)**	77 vs. 21	11 (9–13) vs. 12 (10–17)	**0.038**	1.2 (0.8–1.7) vs. 1.3 (0.9–1.8)	0.21
**IL-18** **(−607 C/A)**	CC	73	11 (9–13)		1.2 (0.8–1.7)	
	CA	20	11 (9–12)		1.2 (0.8–1.6)	
	AA	5	11 (9–12)	0.81	1.3 (0.9–1.8)	0.74
**IL-8** **(−251 A/T)**	TT	51	10 (9–13)		1.1 (0.7–1.5)	
	AT	36	11 (9–13)		1.3 (0.9–1.8)	
	AA	11	12 (10–14)	0.29	1.8 (1.2–2.4)	**0.047**
**IL-8** **(+781 C/T)**	CC	44	10.5 (9–14)		1.2 (0.8–1.6)	
	CT	14	11 (9–13)		1.2 (0.9–1.7)	
	TT	40	11 (9–13)	0.47	1.3 (0.9–1.7)	0.61

Continuous variables are presented as median (IQR). Comparisons between genotype groups were performed using the Kruskal–Wallis test, and dominant model comparisons using the Mann–Whitney U test.

**Table 4:** Multivariable Regression Analyses of Cytokine Variations and MRI Disease Activity.

Predictors of High Baseline Lesion Burden	OR	95% CI	*p* Value	Predictors of Annual Lesion Development	β	95% CI	*p* Value
**IL-18 (−137) C allele (GC+CC)**	2.34	1.08–5.09	**0.031**	**IL-8 (−251) AA genotype**	0.42	0.05–0.79	**0.024**
Age	1.02	0.98–1.05	0.21	Age	0.01	−0.01–0.03	0.29
Female sex	0.94	0.43–2.05	0.87	Female sex	−0.05	−0.28–0.18	0.66
Disease duration	1.04	0.99–1.10	0.09	Disease duration	0.02	−0.01–0.05	0.18

Logistic regression was used for baseline lesion burden (≥median), and linear regression was used for annual lesion formation.

## Data Availability

The data presented in this study are available on request from the corresponding author. The data are not publicly available due to ethical reasons.

## Supplementary Materials

The following supporting information can be downloaded at: https://www.mdpi.com/article/10.3390/jcm15093281/s1, Table S1: A: Association Between Cytokine Gene Variations and Disease Severity in MS Patients. B: Association Between Cytokine Gene Variations and Clinical Presentation of MS.

## Abbreviations

The following abbreviations are used in this manuscript:

RT-PCR	Real-time polymerase chain reaction
EDTA	Ethylenediaminetetraacetic acid
MS	Multiple sclerosis
IL-18	Interleukin-18
IL-8	Interleukin-8
RRMS	Relapsing remitting multiple sclerosis
EDSS	Clinical severity
