# Supplementary material for: Differential Roles of IL-18 and IL-8 Gene Variations in Multiple Sclerosis: Associations with Susceptibility and MRI Disease Activity
Source: J Clin Med. 2026 Apr 25;15(9):3281. doi: 10.3390/jcm15093281 (PMC13164207; doi:10.3390/jcm15093281)
Supplement: Supplementary file 1 [file jcm-15-03281-s001.zip › jcm-4260390-supplementary.pdf]

Supplementary Table S1: A: Association Between Cytokine Gene Variations and Disease Severity in MS Patients. B: Association Between Cytokine Gene Variations and Clinical Presentation of MS

Variations	Genotype	n	EDSS median (IQR)	Time to EDSS ≥3 months median (IQR)	Annual relapse rate median (IQR)
**IL-18 (-607 C/A)**	AA	5	1.5 (1.5–1.5)	6 (6–6)	0.16 (0.13–0.33)
	CA	20	3.5 (1.88–5.50)	60 (45–69)	0.21 (0.11–0.27)
	CC	73	3.0 (1.50–5.00)	66 (33–111)	0.21 (0.12–0.33)
	**p value**		0.41	0.33	0.99
**IL-18 (-137 G/C)**	CC	4	4.0 (3.00–4.75)	36 (21–108)	0.15 (0.12–0.19)
	GC	17	4.5 (2.00–5.50)	108 (63–183)	0.26 (0.11–0.50)
	GG	77	3.0 (1.50–4.50)	60 (24–84)	0.20 (0.12–0.30)
	**p value**		0.47	0.07	0.47
**IL-8 (-251 A/T)**	AA	11	4.0 (2.75–5.00)	60 (57–90)	0.16 (0.11–0.26)
	AT	36	3.0 (1.50–5.12)	72 (42–120)	0.22 (0.13–0.33)
	TT	51	3.0 (1.50–4.75)	48 (7.5–105)	0.18 (0.10–0.32)
	**p value**		0.47	0.34	0.48
**IL-8 (+781 C/T)**	CC	44	3.0 (1.50–4.50)	72 (42–114)	0.18 (0.11–0.26)
	CT	14	3.25 (1.50–5.88)	60 (48–120)	0.20 (0.11–0.26)
	TT	40	3.0 (1.50–5.00)	60 (15–78)	0.23 (0.12–0.45)
	**p value**		0.87	0.39	0.29

Variations	Genotype	n	First symptom n (%)	Symptom at diagnosis n (%)	Time to diagnosis (months) median (IQR)
**IL-18 (-607 C/A)**	AA	5	optic 1 (20) / brainstem 2 (40) / supratentorial 2 (40)	optic 1 (20) / brainstem 2 (40) / supratentorial 2 (40)	36 (12–48)

	CA	20	optic 5 (25) / brainstem 6 (30) / spinal 3 (15) / supratentorial 6 (30)	optic 4 (20) / brainstem 7 (35) / spinal 3 (15) / supratentorial 6 (30)	29 (12–60)
	CC	73	optic 18 (25) / brainstem 20 (27) / spinal 9 (12) / supratentorial 26 (36)	optic 16 (22) / brainstem 19 (26) / spinal 10 (14) / supratentorial 28 (38)	12 (6–48)
	**p value**		0.57	0.93	0.10
**IL-18 (-137 G/C)**	CC	4	optic 1 (25) / brainstem 1 (25) / spinal 1 (25) / supratentorial 1 (25)	optic 1 (25) / brainstem 1 (25) / spinal 1 (25) / supratentorial 1 (25)	66 (24–84)
	GC	17	optic 3 (18) / brainstem 6 (35) / spinal 2 (12) / supratentorial 6 (35)	optic 3 (18) / brainstem 5 (29) / spinal 3 (18) / supratentorial 6 (35)	24 (12–60)
	GG	77	optic 20 (26) / brainstem 21 (27) / spinal 10 (13) / supratentorial 26 (34)	optic 17 (22) / brainstem 22 (29) / spinal 9 (12) / supratentorial 29 (37)	24 (12–48)
	**p value**		0.23	0.30	0.46
**IL-8 (-251 A/T)**	AA	11	optic 3 (27) / brainstem 3 (27) / spinal 1 (9) / supratentorial 4 (36)	optic 3 (27) / brainstem 3 (27) / spinal 1 (9) / supratentorial 4 (36)	24 (12–60)
	AT	36	optic 9 (25) / brainstem 11 (31) / spinal 4 (11) / supratentorial 12 (33)	optic 8 (22) / brainstem 10 (28) / spinal 5 (14) / supratentorial 13 (36)	24 (12–48)
	TT	51	optic 12 (24) / brainstem 14 (27) / spinal 7 (14) / supratentorial 18 (35)	optic 11 (22) / brainstem 15 (29) / spinal 6 (12) / supratentorial 19 (37)	12 (6–36)
	**p value**		0.43	0.73	0.54
**IL-8 (+781 C/T)**	CC	44	optic 11 (25) / brainstem 12 (27) / spinal 6 (14) / supratentorial 15 (34)	optic 10 (23) / brainstem 13 (30) / spinal 6 (14) / supratentorial 15 (34)	24 (12–48)
	CT	14	optic 3 (21) / brainstem 5 (36) / spinal 2 (14) / supratentorial 4 (29)	optic 3 (21) / brainstem 4 (29) / spinal 2 (14) / supratentorial 5 (36)	24 (12–60)
	TT	40	optic 10 (25) / brainstem 10 (25) / spinal 5 (12) / supratentorial 15 (38)	optic 9 (23) / brainstem 10 (25) / spinal 5 (12) / supratentorial 16 (40)	24 (12–48)
	**p value**		0.24	0.96	0.76

Categorical variables were compared using the chi-square test, and continuous variables using the Kruskal–Wallis test
